# Close the pack of cigarettes – close the care gap: Reflections after World Cancer Day 2022

**DOI:** 10.18332/tid/148145

**Published:** 2022-05-09

**Authors:** Paweł Koczkodaj, Kara P. Wiseman, Melissa Little, Roger T. Anderson

**Affiliations:** 1 Cancer Epidemiology and Primary Prevention Department, Maria Sklodowska-Curie National Research Institute of Oncology, Warsaw, Poland; 2 Department of Public Health Sciences, School of Medicine, University of Virginia, Charlottesville, United States

**Keywords:** tobacco, cancer prevention, cancer care, world cancer day

**Dear Editor,**

Each year on 4 February we celebrate World Cancer Day. This year’s theme was *‘Close the care gap’*. Moreover, the Union for International Cancer Control (UICC) has highlighted the importance of healthcare disparities by continuing this theme in 2023 and 2024. Undoubtedly, cancer care is one of the most crucial elements of oncology, however, both from a patient and global health perspective it is essential that we broaden the definition of ‘care’ beyond cancer treatment to include gaps in prevention and self-care, particularly in the area of smoking cessation.

As much as 90–95% of all cancer cases are attributable to highly modifiable risk factors including tobacco use, obesity, physical inactivity, poor diet (plus alcohol consumption), and bacterial and viral infections^1^. In the case of cigarette smoking, for example, more than 90% of lung cancer cases in men and up to 80% among women are caused by cigarette smoking^2^.

To illustrate the importance of a broader definition of ‘care’ for this year and future World Cancer Days, we call attention to global data^3,4^ on the population attributable risk fraction (PAF) associated with tobacco, alcohol use, and obesity (Table 1). At a global level, the PAFs for these modifiable risk factors are substantial; however, the PAF medians for tobacco smoking are notably concerning. Therefore, closing the cancer care gap will be impossible without a marked improvement in reducing tobacco use worldwide, requiring a greater investment in public health outreach and preventative services.

**Table 1:** Population attributable risk fraction (PAF) medians for tobacco, alcohol, overweight and obesity associated cancers^3^, number of new cases, percentages of all sites in 2020^4^

*Risk factor*	*Cancer site (ICD code)*	*Median PAF^3^ (%)*		*Number of new cases and percentages of all sites^4^*	
		*men*	*women*	*n*	*%*
Tobacco smoking	Lung (C-34)	80.5	58.4	2261419	11.4
	Larynx (C-32)	75.2	62.3	184615	1.0
	Esophagus (C-15)	51.1	32.6	604100	3.1
	Oral cavity and pharynx (C-00-C-14)	50.0	18.2	377713	2.0
	Bladder (C-67)	40.7	20.5	573278	3.0
	Kidney (C-64-C-66)	26.4	10.7	431288	2.2
	Pancreas (C-25)	25.5	13.0	495773	2.6
	Liver (C-22)	24.6	8.9	905677	4.7
	Stomach (C-16)	20.1	3.2	1089103	5.6
Alcohol consumption	Oral cavity and pharynx (C-00-C-14)	37.7	16.7	377713	2.0
	Liver (C-22)	29.0	13.0	905677	4.7
	Larynx (C-32)	26.6	10.1	184615	1.0
	Esophagus (C-15)	25.3	11.3	604100	3.1
	Colorectal (C-18-C-20)	15.0	4.1	1931590	10.0
	Female breast (C-50)	-	5.6	2261419	11.7
Overweight and obesity	Esophagus (C-15)	29.0	37.0	604100	3.1
	Kidney (C-64)	14.0	24.5	431288	2.2
	Colorectal (C-18-C-20)	11.8	11.6	1931590	10.0
	Gallbladder (C-23)	11.0	42.5	115949	0.6
	Postmenopausal female breast (C-50)	-	10.0	2261419	11.7
	Endometrium (C-54, C-55)	-	36.0	604127	3.1

Within countries, the rates of preventable cancers also follow income or social class gradients and quantify addressable inequities. However, regardless of the base incidence rates or income status of a given country, healthcare systems face similar challenges worldwide, including under-financing, staff shortages, lack of insurance coverage, and lack of health facilities in the closest area. These factors – amplified by the COVID-19 pandemic – have led to limited accessibility of healthcare services aimed at reducing the burden of cancer, including controlling behavioral risk factors such as tobacco use. Relying on a healthcare system as a main pillar of our approach to combat the burden of cancer can turn out to be very risky without a much larger commitment to cancer self-care and preventive services supported by community organizations.

World Cancer Day 2022 provides an excellent opportunity to rethink the term ‘cancer care’. Taking responsibility for our own health becomes an extremely important issue in the fight against cancer. But it is also something we, as individuals, cannot do alone.

## Data Availability

Data sharing is not applicable to this article as no new data were created.
